# Infected Cochlear Implant and Re-implantation in a Pediatric Case

**DOI:** 10.7759/cureus.35613

**Published:** 2023-02-28

**Authors:** Muhammad Fawwaz Meor Abdul Malik, Noor Dina Hashim, Wan Nabila Wan Mansor, Norhaslinda Abdul Gani

**Affiliations:** 1 Otorhinolaryngology - Head and Neck Surgery, Universiti Kebangsaan Malaysia Medical Centre, Kuala Lumpur, MYS; 2 Otorhinolaryngology/Otology, Universiti Kebangsaan Malaysia Medical Centre, Kuala Lumpur, MYS; 3 Otorhinolaryngology - Head and Neck Surgery, Hospital Tuanku Ja'afar Seremban, Negeri Sembilan, MYS

**Keywords:** equipment failures, risk factors, bilateral severe to profound hearing loss, auditory prothesis, cochlear implant

## Abstract

Cochlear implant (CI) surgery is relatively safe, however reports of complications and failure following cochlear implant surgery are higher nowadays due to the increasing number of patients with CI. Herein, we report a case of infected cochlear implant 10 months after surgery. A three-year-six-month-old girl underwent right cochlear implantation for bilateral profound sensorineural hearing loss. From day one until six months after the surgery, it was uneventful and the wound healed well. However, at 10 months post-surgery, she presented with a chronic discharging wound over the previous surgical site. Despite being on IV antibiotics for six weeks and daily dressing, the wound over the implant site keep discharging and eventually the implant was removed two months later. She was later re-implanted with a cochlear implant on the same side at the age of five years 10 months old. Currently, she is showing good speech improvement with the right CI. Her aided hearing threshold is at 30-40 dB at all frequencies.

Early diagnosis is crucial, and the proper course of action should be taken as soon as possible if implant failure is suspected. Prior to implant surgery, any potential risk factors that could lead to implant failure should be identified and addressed appropriately to reduce the risk of an infected cochlear implant.

## Introduction

The most well-known and effective hearing implant for severe to profound sensorineural hearing loss is the cochlear implant (CI). It is a gold standard treatment [1] for bilateral severe to profound sensorineural hearing loss in those who have not benefited from hearing aids and will have a significant positive impact on both pre-lingual and post-lingual deafness patients. Cochlear implant surgery is relatively safe; however, surgery and device-associated complications are inevitable in a few cases [2,3]. Here, we would like to report a case of cochlear implant failure after 10 months of surgery and discuss the common complications associated with implant failure.

## Case presentation

**Figure 1:**
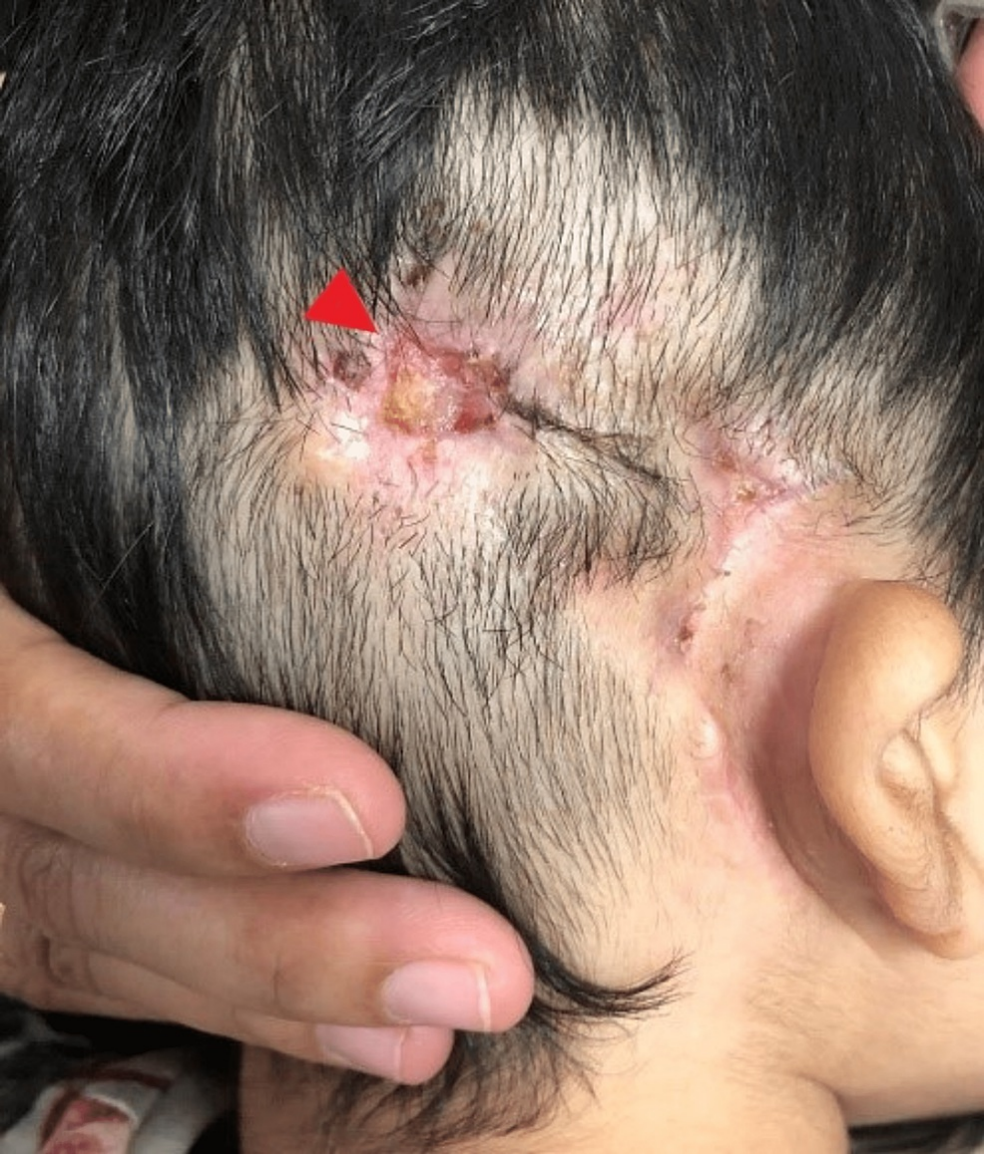
Six months after surgery, a healed scar over the right postauricular region and thinned skin flap over the receiver site with scab (arrowhead).

**Figure 2:**
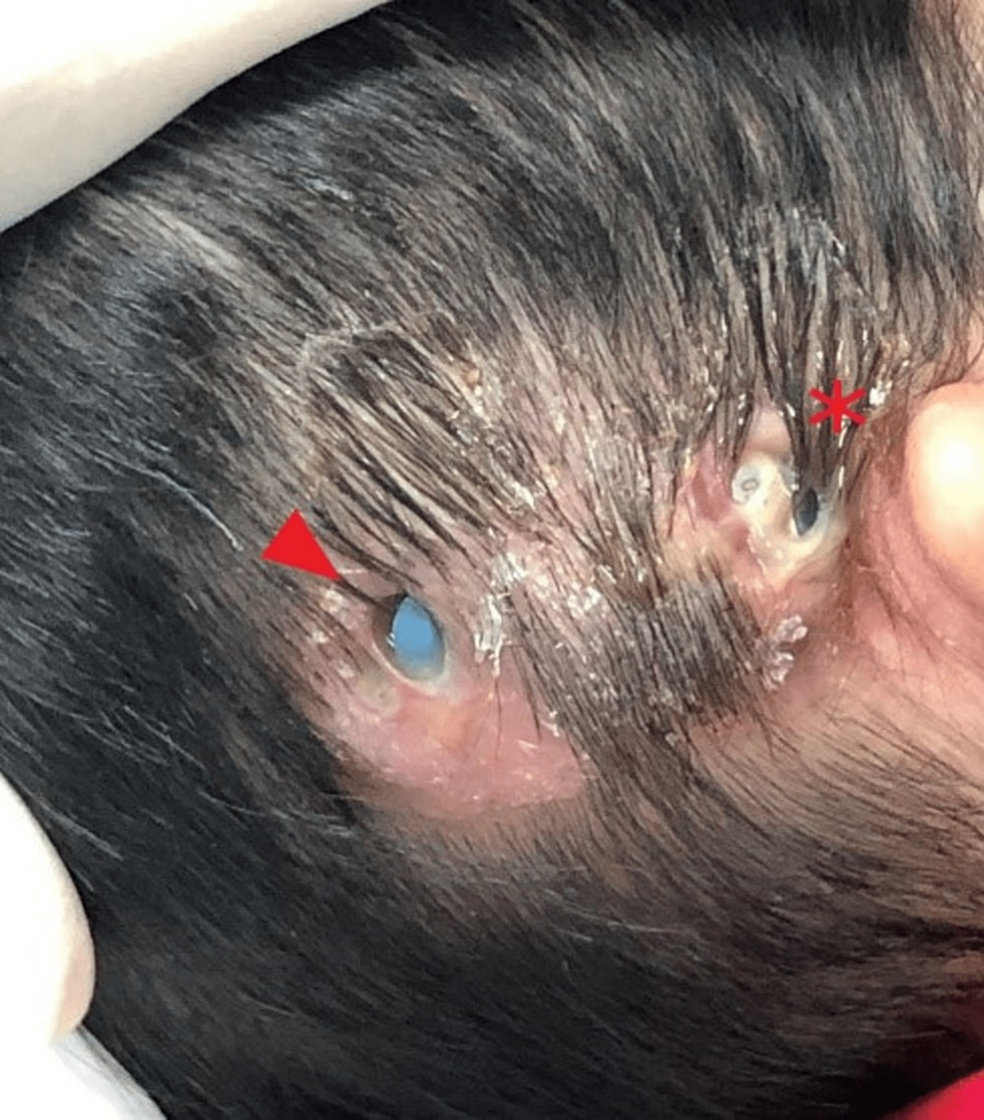
Surgical wound after 10 months showing two wounds breakdown over the postauricular wound (asterisk) and receiver (arrowhead) site with seropurulent discharge exposing the implant.

A three-year-six-month-old girl underwent right cochlear implant for bilateral profound sensorineural hearing loss. There was no allergic history or skin pathology that could increase the risk of skin reaction. She had a history of persistent right middle ear effusion which required myringotomy and ventilation tube insertion one year prior to CI surgery. The ventilation tube was self-extruded at six months post-surgery and middle ear effusion was resolved. The right cochlear implant surgery went well with no immediate intraoperative or postoperative complications. Intraoperative findings showed no edema and granulation tissue in the middle ear and mastoid cavity. On followup, her speech development was good, aided hearing threshold ranges from 25 to 35 dB across all frequencies. Six months after surgery, the right postauricular scar healed, however, the skin flap over the receiver site appeared thin and was covered with a thick scab without discharge. (Figure 1). She was closely monitored. At 10 months post-surgery, there were two discharging open wounds over the receiver site and right post auricular scar. The implant was partially exposed (Figure 2). Despite daily dressings over the wound and a six-week course of broad-spectrum IV antibiotics, she had shown no signs of wound healing, suggesting that the removal of the implant was imminent.

Subsequently she underwent removal of the implant at the age of four years five months old. During the surgery, granulation tissue was observed in the mastoid cavity and previous posterior tympanostomy site. The implant electrode was in-situ within the cochlea which was then removed followed by reinsertion of a dummy electrode. The infected wound and tissue are debrided and skin closed primarily. The previous implant was not examined for culture. However, soft tissue biopsy taken from post auricular incision and implant site revealed foreign body granuloma and grew Staphylococcus aureus. A two-week course of cloxacillin was given to counter the microorganism. After CI removal, the wound healed well. She was reimplanted back with cochlear implant at the age of five years 10 months old (17 months after implant removal) and it was uneventful surgery and currently under regular follow-up in the clinic and showing good progress of speech and good hearing with CI. 

## Discussion

In general, the overall incidence of cochlear implant failure was reported between 1.7 - 3.3 %. There are three categories of CI failures: hard failure, soft failure and medical failure [4,5]. Hard device failure includes a malfunctioning device, such as complete failure of the internal device which can be seen in post head trauma cases. It is confirmed by the presence of auditory input interruption combined with an abnormal integrity test [4,5]. All medical issues, such as infection, flap necrosis, trauma, and electrode migration that are unrelated to electronic issues are considered medical failures [5]. The incidence of medical failure varies widely and is reported between 1.4% - 8.2% [6]. 

Flap infection is one of the medical complications that occur after CI which can be classified into flap seroma or hematoma around the receiver-stimulator, skin flap infection or necrosis with development of granulation tissue over the wound, or skin flap rupture with implant exposure and extrusion [7]. In a study done by Kim et al., flap-associated complications following CI surgery were the second most common complication after device failure which involves 0.4% of the patients [3]. As in this case, there is a high possibility of skin flap necrosis or infection over the receiver site may occur perhaps due to underlying middle ear effusion (MEE). However, previous studies reported that CI surgery on MEE patients has possible operative difficulties due to bleeding from inflamed edematous tissue but complication rates post-surgery among patients with or without MEE are nearly the same [8,9]. Despite that, Cunningham et al. recommend that patients undergoing CI with significant history of ear disease should be counselled on possible risk of CI failure postoperatively and seek early treatment if symptoms or signs arise and it should be treated with aggressive antibiotic treatment [10].

CI failure is seen more frequently among the paediatric age group and many previous publications attribute it due to increase rate of ear infection, head trauma and skull bone growth as potential risk factors for CI failure [11,12]. Most of the infected CI cases occurred secondary to biofilm-related infections and resistance to the antimicrobial therapy given [7]. The most common pathogen isolated from the wound and implant is Staphylococcus aureus, which is similar in our case. Other possible pathogens are Escherichia coli, Group A beta-hemolytic Streptococcus, Klebsiella pneumoniae, and Pseudomonas aeruginosa [10]. Treatment for an infected cochlear implant is similar to other infected biomedical devices, in which the principle is to remove the implant and culture-directed antimicrobial therapy [6]. However, the current recommendation favors a conservative approach [10]. If the removal of the implant is imminent, Cunningham et al. recommend leaving the electrode inside the cochlea to prevent risk of cochlear ossification which can compromise future attempts at CI electrode insertion [10]. Timely surgical revision is required for skin flap defect, but if it fails, judicious implant removal and contralateral or ipsilateral CI should be considered.

## Conclusions

Complications after CI surgery are rare, but the increasing number of reported cases will make surgeons more vigilant to detect early or late complications. Any potential risk factors that may lead to implant failure should be identified and treated early, in particular MEE and acute otitis media (AOM), which are common in the pediatric age group. Cochlear implant reimplantation is necessary for children with profound sensorineural hearing loss, but the risks and benefits should be weighed before surgery. 
